# Intermediate Syndrome Due to Organophosphate Poisoning: A Case Report

**DOI:** 10.7759/cureus.39368

**Published:** 2023-05-23

**Authors:** Shubhangi Setia, Anjalee Chiwhane

**Affiliations:** 1 Medicine, Jawaharlal Nehru Medical College, Datta Meghe Institute of Higher Education and Research, Wardha, IND

**Keywords:** suicide attempt, acute pesticide poisoning, poisoning, opp, organophosphate, intermediate syndrome, insecticide, farmer's suicide, atropine, acetylcholinesterase

## Abstract

Organophosphates, also known as phosphate esters, are a category of pesticide compounds that function by indirectly inhibiting the activity of an enzyme called acetylcholinesterase (AChE). AChE is responsible for breaking down acetylcholine (ACh) at the neuromuscular junction into acetic acid and choline. These compounds cause various clinical presentations upon acute toxicity, among which intermediate syndrome (IMS) exhibits an unpredictable course. This report describes the case of a farmer who ingested monocrotophos and ethanol in a suicide attempt, leading to a prolonged stay in the hospital and invasive ventilation, along with complications including ventilator-associated pneumonia. The patient received a total of 9000 mg of atropine over his 14-day hospitalization period.

## Introduction

The estimated number of pesticide self-poisoning deaths is more than 14 million from the 1960s to 2018 [1]. Pesticide poisoning is responsible for half a million fatalities annually in Asia and the Western Pacific, with organophosphate (OP) compounds being the primary cause [2].

In a country like India, where agriculture is a critical industry, farmer suicide is a pressing concern. Due to the ready availability of these compounds, it is not uncommon to witness their misuse. Each year, approximately 76,000 cases of OP poisoning are reported in India. Our tertiary care institute commonly handles such cases due to its proximity to rural areas, where most of the population are farmers. Routes of exposure to these compounds include ingestion, skin absorption, and inhalation [1]. Acute exposure to OP can lead to muscarinic symptoms such as diarrhea, urination, lacrimation, miosis, bronchoconstriction, nausea, vomiting, and muscle weakness. Chronic exposure, on the other hand, can result in more severe symptoms such as loss of coordination, impaired cognition, and speech loss. OP compounds include malathion, parathion, diazinon, phosmet, and methyl parathion. The use of monocrotophos, an OP insecticide, has been prohibited in many countries, such as the United States, Europe, and India, due to its potential health hazards [2]. However, in some regions where its regulation is not stringent, it is still accessible.

A patient experiencing OP poisoning may exhibit three toxic effects phases, namely acute cholinergic crisis, intermediate syndrome (IMS), and delayed distal polyneuropathy [3]. IMS is a dangerous complication of OP intake that usually appears 24 to 96 hours after apparent recovery from the acute cholinergic syndrome. This is because of the prolonged inhibition of acetylcholinesterase (AChE), leading to heightened stimulation of nicotinic receptors by acetylcholine, resulting in muscular weakness and paralysis. Around 20% of patients who consume OP orally develop IMS [4,5]. The initial symptoms include action tremors and pharyngeal weakness, leading to pooling secretions. Later, there is difficulty flexing the neck, proximal limb muscle weakness, and respiratory paralysis. IMS is believed to occur when at least 80% of the synaptic AChE is inhibited [6]. The duration of IMS symptoms may vary from two to 18 days [7]. Since the pathophysiology of IMS remains unclear, supportive therapy is the primary treatment option [3]. According to a recent study, the mortality rate for IMS patients with respiratory failure was 28.4% [8].

This report introduces a case involving a 22-year-old male who consumed monocrotophos with ethanol, subsequently developed ventilator-associated pneumonia, and endured a protracted recovery period due to IMS.

## Case presentation

A 22-year-old male patient of Indian descent, working as a farmer, consumed 500 ml of monocrotophos and an undisclosed quantity of ethanol around 8:30 PM on January 2, 2023. He encountered two episodes of vomiting, characterized by the expulsion of watery fluid mixed with food particles. Initial medical attention was administered at a private hospital, where the patient underwent intubation and was connected to mechanical ventilation. At 3:30 AM on January 3, 2023, he was referred to the emergency department of our institute.

Upon admission, the patient was admitted and underwent gastric lavage. Intravenous administration of pralidoxime was initiated, with four daily doses on both day one and day two. Additionally, an injection of atropine 2cc was administered. The patient presented with a pulse rate of 140 beats per minute, a blood pressure reading of 180/90 mm Hg, a respiratory rate of 25 breaths per minute, and a high fever. The patient's medical history did not indicate any significant past illnesses. The Glasgow Coma Scale score was 8 out of 15, and the patient's pupils were constricted and unresponsive in both eyes. Deep tendon reflexes were sluggish, with a power grade of 3/5 in all four limbs, but there was no sensory impairment. Generalized muscle twitches were observed. The patient exhibited weak head-holding, and trauma was ruled out as a cause. Oxygen saturation levels were measured at 98%, and arterial blood gas (ABG) analysis revealed metabolic and respiratory acidosis. An X-ray conducted during admission indicated right middle lobe pneumonia, possibly resulting from aspiration. Tachycardia was observed, while no enlargement of organs was detected. Invasive mechanical ventilation was initiated in response to the abnormal ABG findings. The patient's ABG values showed a mixed acidosis with a blood pH of 7.288 and partial pressure of carbon dioxide (pCO2) of 41.9, alongside right middle-lobe pneumonia.

**Figure 1:**
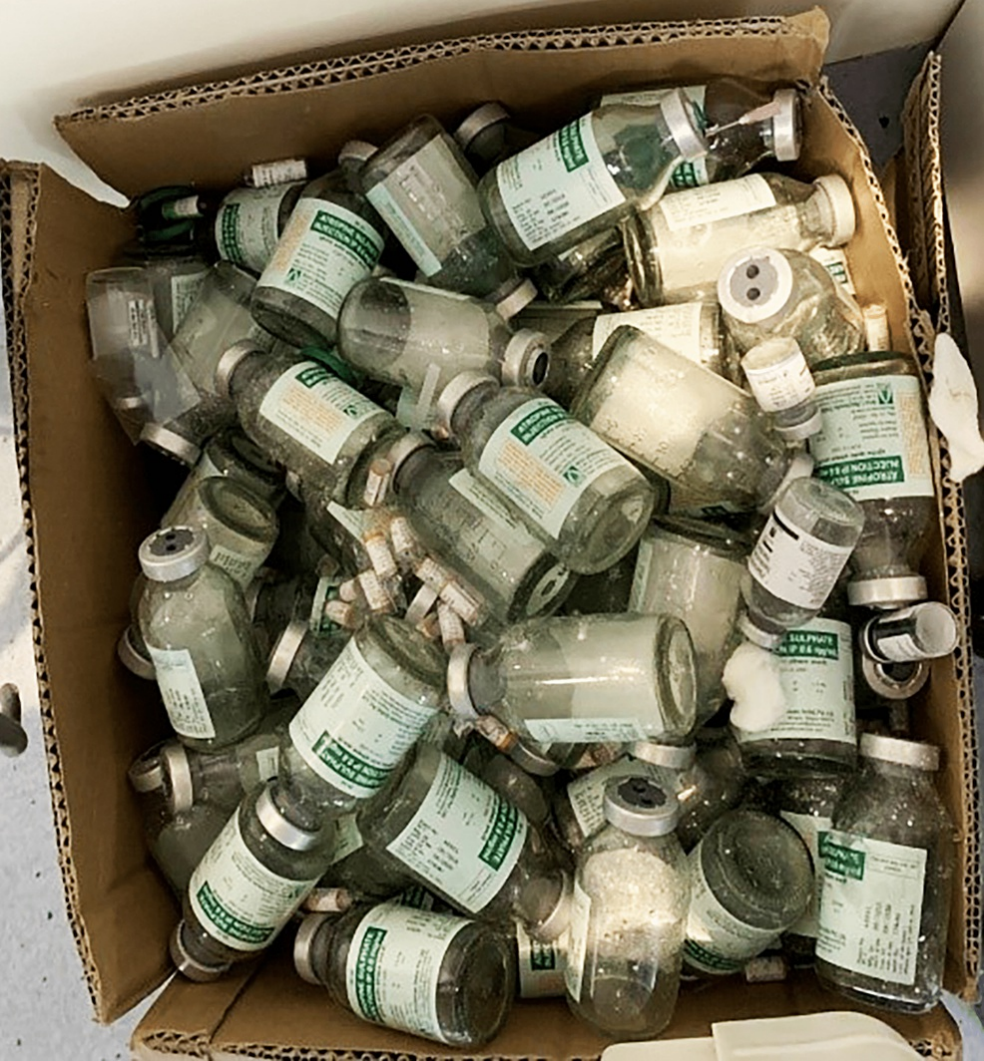
Total vials of atropine administered over 15 days which was an astounding dose of 1500ml

**Figure 2:**
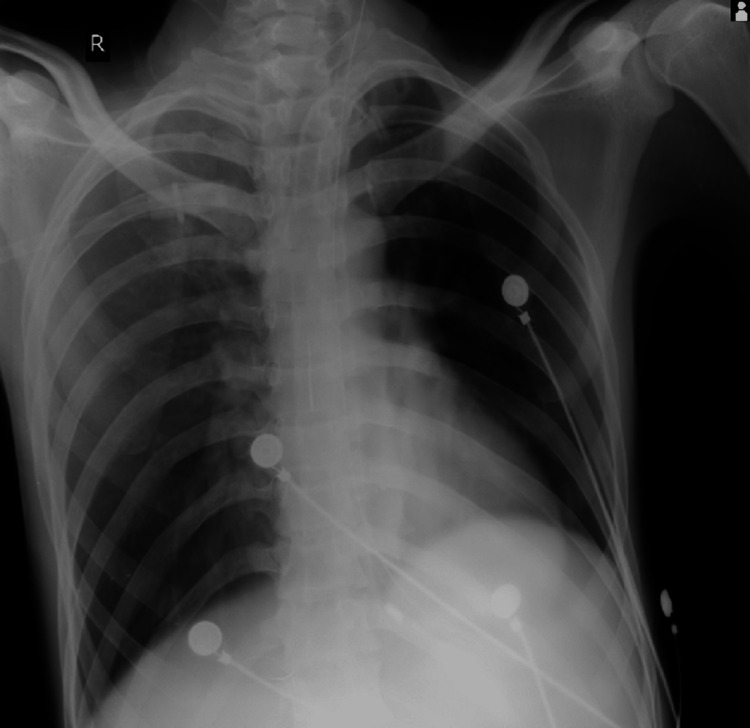
X-ray of January 5, 2023, showing patch in middle lobe with the possibility of ventilator-associated pneumonia

**Figure 3:**
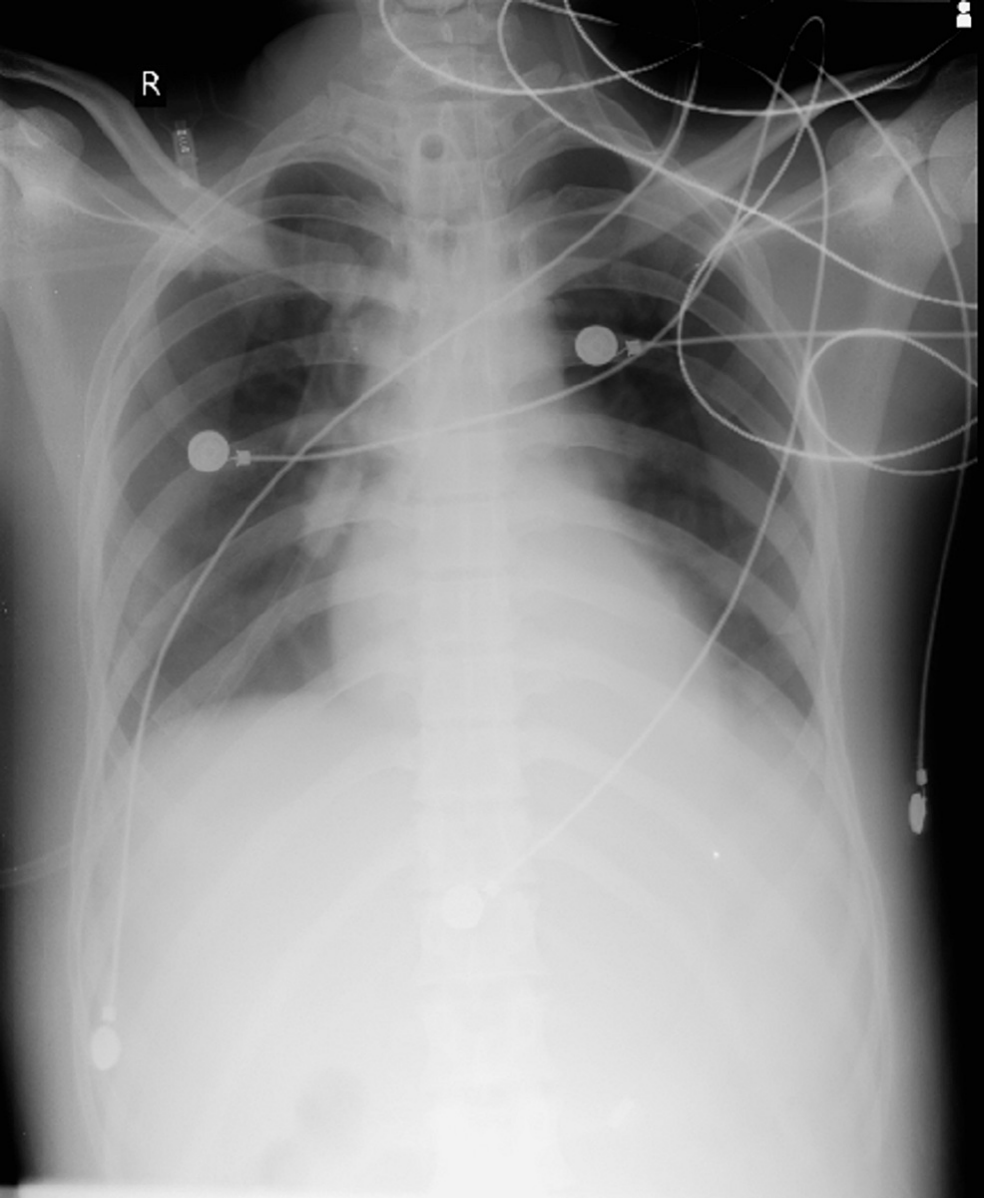
X-ray of January 15, 2023, showing clearing of middle patch

The hemoglobin level was found to be 8.7g/dL, as revealed by the test results. The peripheral smear showed red blood cells (RBCs) that were predominantly normocytic and mildly hypochromic. Platelet levels were adequate based on the smear analysis. No haemoparasites were observed. The total leukocyte count was within the normal range. However, the serum AChE levels on day 2 were significantly low, measuring 127.7 U/L, whereas the normal range is 4900-11900 U/L. On day 4, the levels increased to 251.8 U/L. Culture and sensitivity tests conducted on blood, urine, and endotracheal tube secretions produced normal results. Moreover, blood cultures obtained upon admission and 48 hours later showed no signs of bacterial growth. However, sputum samples collected on the morning of day two indicated the presence of Gram-negative bacilli, specifically *Acinetobacter *and *Pseudomonas *species. Four days after admission, the patient was administered antibiotics based on sensitivity testing, namely IV colistin and ceftriaxone, as well as atropine and pralidoxime. Notably, the patient required an exceptionally high dosage of atropine (9000 mg) over a period of 14 days (Figure 1). The patient's blood test results displayed significant variability, with platelet and white blood cell (WBC) counts showing fluctuating patterns throughout the hospital stay. On January 15, 2023, the X-ray revealed clearance in the central region of the right lung, and the patient exhibited improved limb movements from January 2023 onwards until February 5, 2023 (Figures 2-3).

On February 5, 2023, the patient's family members made a request for discharge against medical advice, which was subsequently approved by the authorities. Upon the patient's discharge, his body temperature was normal, with a blood pressure reading of 120/80 mmHg and a pulse rate of 80 beats per minute. The patient exhibited a Glasgow Coma Scale score of 15 out of 15, indicating full consciousness, and had grade 3 out of 5 power in both upper and lower limbs, with the ability to hold his head up. The hemoglobin level measured 8.2 grams per deciliter, while the WBC and platelet counts appeared normal, alongside unremarkable X-ray results. ABG values fell within the normal range, and the remaining blood chemistry tests yielded normal results. Despite these positive indicators, the patient still required Ryle's tube feeding due to his inability to swallow and remained reliant on external support when sitting.

## Discussion

The presented case describes a 22-year-old male farmer who ingested monocrotophos, an OP compound commonly used in agriculture, and an undisclosed amount of ethanol. OP poisoning is a severe medical emergency that can lead to life-threatening complications. The initial symptoms observed in the patient, such as vomiting and subsequent respiratory distress requiring intubation and mechanical ventilation, were consistent with OP toxicity [9].

Upon admission, the patient exhibited various clinical manifestations associated with OP poisoning. These included high fever, tachycardia, constricted and unresponsive pupils, and neurological deficits, as evidenced by a Glasgow Coma Scale score of 8 out of 15. The patient also displayed weakness, generalized muscle twitches, and sluggish deep tendon reflexes. These findings align with the cholinergic crisis induced by OPs [10].

Laboratory investigations revealed significant abnormalities consistent with OP poisoning. The patient exhibited metabolic and respiratory acidosis as indicated by ABG analysis. The reduced serum AChE levels confirmed the inhibition of this enzyme by OPs, further supporting the diagnosis [10]. Additionally, the patient developed right middle-lobe pneumonia, likely resulting from aspiration during the initial vomiting episodes. This complication highlights the importance of maintaining airway protection and considering prophylactic antibiotic therapy in patients with OP poisoning who are at risk for aspiration [11].

Treatment for OP poisoning involves prompt administration of specific antidotes, supportive care, and management of complications. The patient received pralidoxime, an AChE reactivator, and atropine, an anticholinergic agent. Pralidoxime reverses AChE inhibition, while atropine helps counteract the excessive cholinergic stimulation [10]. The high dosage and prolonged duration of atropine administration, in this case, might reflect the severity of the patient's cholinergic crisis. In addition to the acute complications of OP poisoning, the patient developed secondary infections, specifically *Acinetobacter* and *Pseudomonas *pneumonia. This emphasizes the increased susceptibility to nosocomial infections in patients with compromised respiratory function and prolonged hospital stays [12].

During the hospital course, the patient's laboratory parameters, including platelet and WBC counts, showed fluctuations, indicating the systemic impact of OP toxicity. The gradual improvement in limb movements and radiographic findings over time suggested a positive response to treatment and the patient's ability to recover from the toxic effects of OPs [10].

This case report underscores the importance of prompt recognition and appropriate management of OP poisoning. It also highlights potential complications, such as aspiration pneumonia and nosocomial infections, that can arise during treatment. Further studies and more extensive case series are warranted to enhance our understanding of the optimal management strategies and long-term outcomes in patients with OP poisoning.

## Conclusions

We have filed this case report based on the significant dosage of atropine that was administered and the prolonged duration of ventilation and hospital stay that became necessary. Despite receiving extensive medical care, the patient's incapacitation underscores the urgent necessity for a specialized rehabilitation center that can aid individuals in recovering their self-sufficiency. Furthermore, it is crucial to establish stringent regulations governing the sale and accessibility of toxic substances to the general population. The implementation of laws aimed at preventing the illicit use of such compounds is of utmost importance.
